# Reconstruction of acetabular defects greater than Paprosky type 3B: the importance of functional imaging

**DOI:** 10.1186/s12891-021-04072-4

**Published:** 2021-02-20

**Authors:** Anna Di Laura, Johann Henckel, Elisabetta Dal Gal, Mohammed Monem, Maria Moralidou, Alister J. Hart

**Affiliations:** 1 The Royal National Orthopaedic Hospital, Brockley Hill, Stanmore, London, HA7 4LP UK; 2 Institute of Orthopaedics and Musculoskeletal Science, University College London, London, UK; 3 Department of Mechanical Engineering, University College London, London, UK

## Abstract

**Background:**

3D Surgical planning has become a key tool in complex hip revision surgery. The restoration of centre of rotation (CoR) of the hips and leg length (LL) are key factors in achieving good clinical outcome. Pelvic imaging is the gold standard for planning and assessment of LL. We aimed to better understand if 3D planning is effective at equalising LL when large acetabular defects are present.

**Materials and methods:**

This was a prospective case study of 25 patients. We report the analysis of pre-operative LL status and planned LL restoration measured on CT, in relation to the achieved LL measured post-operatively in functional, weight bearing position. Our primary objective was the assessment of restoration of CoR as well as the anatomical and functional LL using biplanar full-length standing low-dose radiographs; our secondary objective was to evaluate the clinical outcome.

**Results:**

Pre-operative intra-pelvic discrepancy between right and left leg was a mean of 28 mm (SD 17.99, min = 3, max = 60 mm). Post-operatively, the difference between right and left vertical femoral offset (VFO), or CoR discrepancy, was of 7.4 mm on average, significantly different from the functional LL discrepancy (median = 15 mm), *p* = 0.0024. Anatomical LLD was a median of 15 mm. In one case there was transient foot drop, one dislocation occurred 6 months post-operatively and was treated by closed reduction, none of the patients had had revision surgery at the time of writing. Mean oxford hip score at latest follow up was 32.1/48.

**Discussion:**

This is the first study to investigate limb length discrepancy in functional position after reconstruction of large acetabular defects. We observed that VFO is not an optimal surrogate for LL when there is significant bone loss leading to length inequality, fixed flexion of the knee and abduction deformity.

**Conclusions:**

Although challenging, LLD and gait abnormalities can be greatly improved with the aid of an accurate surgical planning. Surgeons and engineers should consider the integration of EOS imaging in surgical planning of reconstruction of large acetabular defects.

## Introduction

Managing large acetabular defects remains a challenging task in revision total hip arthroplasty (THA) [1–4], usually correlated with mechanical deficiency [2]. Goals include reconstructing bone morphology and optimal positioning of the acetabular component to restore centre of rotation (COR) and leg lengths (LL) [5].

Leg length discrepancy (LLD) is associated with a number of pathologies including gait and posture abnormalities [6–8] arthritis of lower limbs and lumbar spine [7] as well as sciatic nerve damage [9]. Although a number of studies have reported on LL restoration following primary THAs [10–14], LL following complex acetabular reconstruction has not been largely addressed.

In case of significant bone loss, CT-based planning is essential to restore the CoR [15]. In planning revision surgery, the patients are CT scanned in supine position [16]; due to the limited field of view, vertical femoral offset (VFO) is used as a surrogate measure for LL. This eliminates key information with regards to functional LL. Low-dose biplanar radiography (LDBR) offers the possibility of upright full length imaging and 3D reconstruction of the limbs to accurately measure LL [17].

We aimed to better understand if CT planning is effective at equalising the limbs when large acetabular defects are present. Our primary objective was to measure the achieved differences in right and left CoR (difference in VFO) as well as functional and anatomical LLD from post-operative standing EOS imaging. Our secondary objective was to evaluate clinical outcomes including OHS, walking status, dislocation rate, revision rate, nerve injury.

## Materials and methods

### Study design and outcome measures

Twenty-five patients with large acetabular defects were a candidate for receiving a custom 3D printed acetabular cup. All cases had radiographic evidence (confirmed on CT and plain radiograph) of at least a Paprosky type 3B acetabular defect, including discontinuities.

**Fig. 1:**
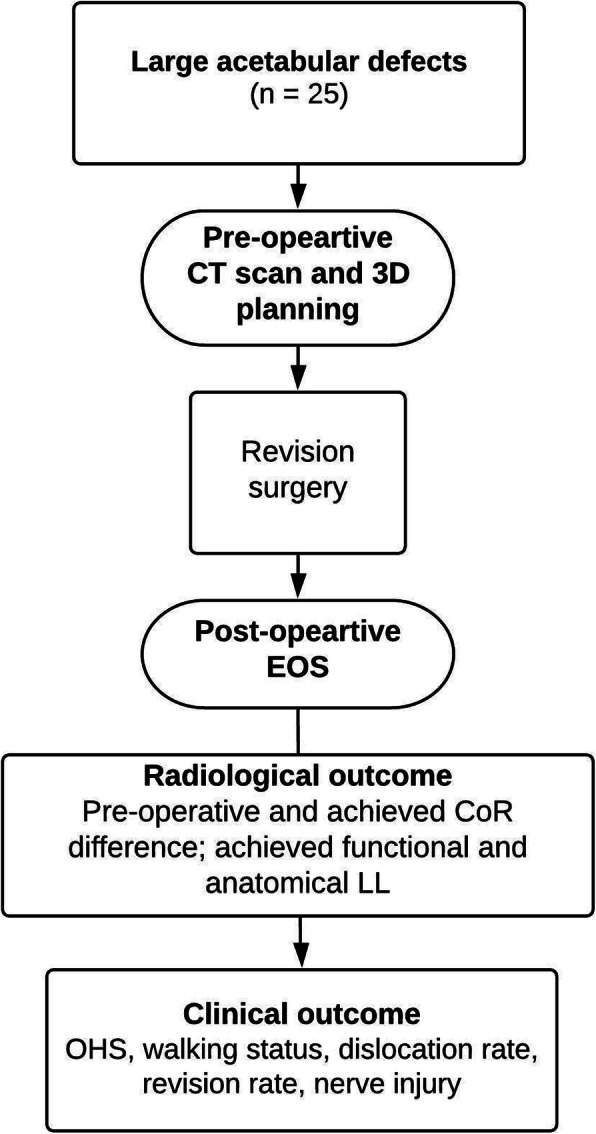
Flowchart of the study design

The patients were post-operatively evaluated using standing 3-dimensional (3D) imaging system (EOS; EOS Imaging SA, Paris, France) at the time of manuscript writing and were therefore included in the study. We report the analysis of pre-operative LLD status and planned CoR restoration in relation to the achieved LL measured post-operatively in functional, weight bearing position, Fig. 1.

There were 16 females and 9 males; mean age at surgery was 67 years (range 49–90).

Patient demographics are displayed in Table 1.

**Table 1:** Patients’ characteristics

Case	BMI	Op #^**a**^	Pre-operative walking aids	Reason for revision
**1**	22	1	Frame with wheels	Loose right total hip replacement with massive bony defects in the acetabulum
**2**	25	2	Walking stick and crutches	Loosening of revision hip arthroplasty
**3**	32	2	Bilateral underarm crutches	Pelvic discontinuity following revision left hip repl.
**4**	30	3	Frame	Dislocated revision right total hip with a constrained liner. Periprosthetic type C fracture, managed by open reduction internal fixation with distal femoral locking plate
**5**	31	2	2 crutches	Massive acetabular defect and loose hip replacement. High CRP with negative aspirate
**6**	32	3	Wheelchair	Failed MoM implant, subsequent loosening and infection of the THR
**7**	26	Multi^b^	2 crutches	Multiple hip operations, severe mechanical symptoms
**8**	48	2	Wheelchair	Failed replacement with acetabular migration
**9**	29	2	None	Failure of MoP hip replacement with superior migration of the hip
**10**	23	2	Wheelchair	Loose R hip replacement with inflammatory pseudotumor
**11**	37	Multi^b^	Walking crutches	Loose left long stem, loose acetabulum
**12**	26	1	Bilateral crutches	Loose R acetabulum MoP replacement with subluxation
**13**	23	1	None	MoM hip resurfacing with high metal ions levels and bone and soft tissue damage
**14**	27	2	Bilateral crutches	Failed MoP L hip with superior migration of cup and stem, pelvic discontinuity
**15**	26	0	Walking crutches	Severe joint degradation. Presence of massive fractured bone protrusion below acetabulum
**16**	26	2	Wheelchair	Failed implant and infection
**17**	30	2	Frame with wheels	Infection and pelvic discontinuity
**18**	22	1	Bilateral crutches	Acetabular cup failure following primary R THA
**19**	26	0	None	Hip Osteoarthritis secondary to acetabular fracture
**20**	23	2	Walking trolley	Catastrophic failure of revision right hip replacement
**21**	30	4	Wheelchair	Loosening and infection
**22**	27	1	Walking stick	Loosening of acetabular component and significant osteolysis
**23**	22	1	Walking stick	MoM modular hip replacement with pseudotumor
**24**	26	Multi^b^	Frame	History of recurrent dislocation and infection
**25**	25	Multi^b^	Wheelchair	Multiple severe infections and metallosis from THA

^a^ Op #, total number of hip replacements including one in study. MoM, Metal on Metal; MoP, Metal on Polyethylene; R, Right; L, Left

^b^ Multi, no data on total number of hip replacements available greater than 2 known.

Component design and pre-operative planning were undertaken with close collaboration between surgeons and engineers. Continued follow up (median 37 months, range 20–52) was performed to monitor clinical outcome.

The outcome measures were: 1) Pre-op Vs achieved VFO, 2) Post-operative anatomical and functional LL 3) OHS, walking status, dislocation rate, revision rate, nerve injury.

### Pre-operative planning

The patients underwent metal artefact reduction sequencing CT scanning of their whole pelvis. All patients were imaged with a Siemens SOMATOM® Definition AS+ 128 slice CT scanner. Images were acquired at 100 kV, 100 mAs [21]. Data was saved as DICOM files, anonymised and provided to the manufacturer, via a secure dedicated portal, to be included into a specific workflow for implant design.

Once approved, the customised implants were produced using electron beam melting EBM additive manufacturing with regions of trabecular titanium to promote osteointegration [18]. Intra-operatively, plastic models of the patient’s anatomy, the bespoke implant and the drill guides were manufactured using 3D printing and sterilised for intraoperative use.

Data from CT scans was used for the accurate assessment of the centre of rotation of the failed hip. The difference in VFO between the right and left side was used as surrogate measure for LL on pelvic CT scans as the full leg is not imaged. These measurements informed on the pre-operative LL status.

**Fig. 2:**
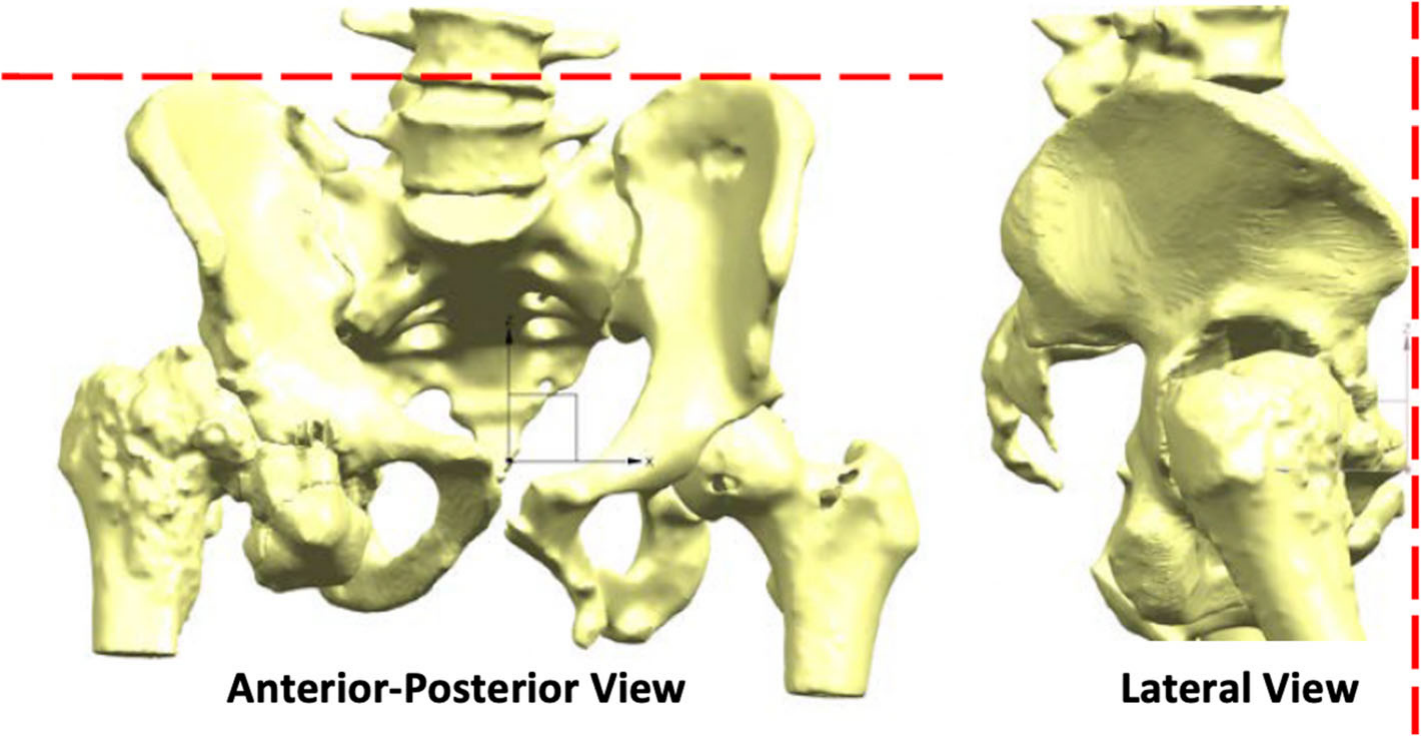
Anteroposterior and lateral views of CT-reconstructed anatomy oriented with 0° pelvic tilt and rotation. The AnteroSuperior Iliac Spines (ASIS) and the pubic symphysis (PS) lie on the same plane

LL from 3D CT reconstruction was considered the distance between left and right hip joint centre of rotation when the pelvis has a sagittal tilt of 0°(the anterior pelvic plane corresponds to the coronal plane of the body), Fig. 2.

Designing the customised titanium implant involved filling the defect with porous titanium, assuring fixation with structural titanium and screw holes and determining the optimal location of centre of rotation so that to restore optimal biomechanics.

**Fig. 3:**
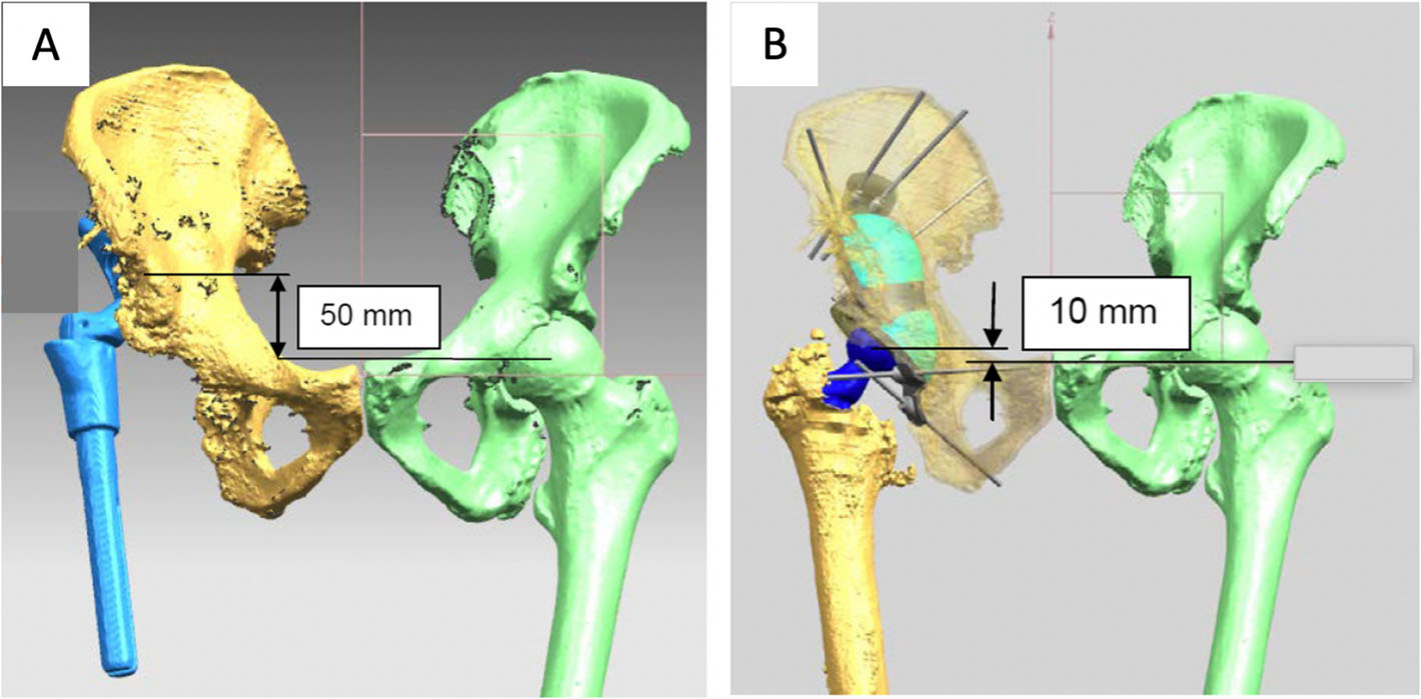
Example of CT surgical plan where complete equalization of left and right centre of rotation was not the goal, a residual 10 mm difference between right and left hip was planned. Decision was made considering the anatomical characteristics of the patients alongside with the design of the bespoke implant to be inserted in the acetabular cavity

Equalization of left and right centre of rotation was not always the goal. Decision was made on a case-basis considering the bone stock and anatomical characteristics, Fig. 3.

### Surgical procedure

Surgical procedures were conducted by one senior orthopaedic surgeon who was sometimes accompanied by another senior orthopaedic surgeon and/or senior vascular surgeon. The key steps in surgery were: an extensive posterior approach, reaming of non-viable bone as per plan, trial with 3D printed plastic implant model and fixation of 3D printed titanium implant with screws and patient specific drill guides. 3D printed patient anatomy helped with appreciating the size of the defect, assisting with surgical exposure, guiding surgical orientation, preparing the bone.

### Post-operative radiological and clinical outcome

EOS scans were taken post-operatively. The simultaneous biplanar acquisition was used to perform stereoradiographic 3D modelling of each lower extremity using specialized software (sterEOS 3D; EOS Imaging SA) [19]. Bilateral VFO as well as full leg segments were measured on EOS scan post-operatively.

VFO was measured as the vertical distance between the joint CoR and the inter-ischial tuberosity line [20, 21]. The difference between right and left VFO was used to measure LLD, as conventionally done when full limb length scans are not available.

**Fig. 4:**
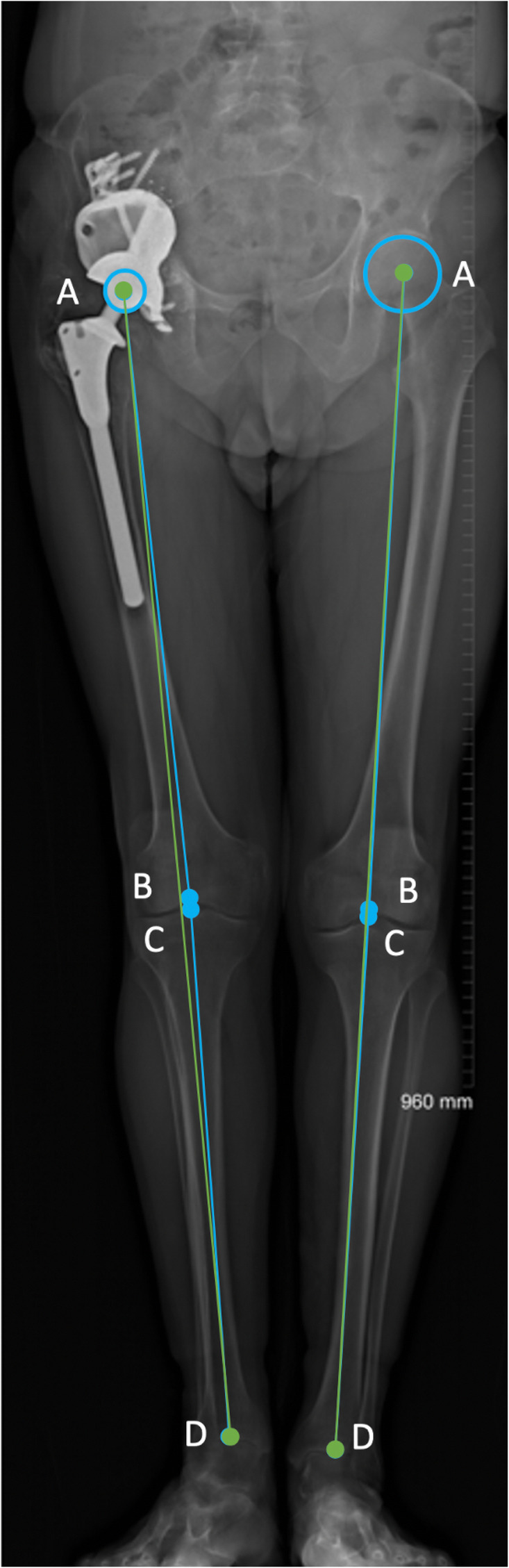
Anatomic vs functional length. (1) Anatomical femoral length: distance between the centre of the femoral head (**a**) and the centre of the trochlea (**b**). (2) Anatomical tibial length: distance between the centre of the tibial spine (intercondylar eminence) (**c**) and the centre of the ankle joint (**d**). (3) Functional length: distance between the centre of the femoral head to the centre of the ankle joint (AD). (4) Anatomical length: sum of the anatomical femoral and tibial lengths (AB + CD)

Moreover, full length measurements of leg length were also taken, the following definitions were used. Anatomical femoral length: distance between the centre of the femoral head (native or implant) and the centre of the trochlea. Anatomical tibial length: distance between the centre of the tibial spine and the centre of the tibial plafond. Functional leg length: distance between the centre of the femoral head (native or implant) to the centre of the ankle joint. Anatomical length: sum of the anatomical femoral and tibial lengths, Fig. 4.

VFO and LL measurements were performed separately by two examiners to test reproducibility.

Follow-up of patients was performed by the senior authors to monitor for complications. Post-operative walking status and oxford hip scores [22] were recorded during latest follow up (20 to 52 months, median 37 months).

### Statistical analysis

Statistical analyses were performed using SPSS® Statistics Version 24 (IBM Corp., Armonk, NY, USA). Intraclass correlation coefficient (ICC) tested the reproducibility of the method of analysis performed by the two examiners. We considered an ICC of > 0.90 as high, between 0.80 and 0.90 as moderate, and < 0.80 as insufficient. Normal distribution of the values was checked by means of the D’Agostino-Pearson omnibus normality test for each series of measurements. For data with normal distribution, paired Student’s t-test was used for analysis. For data without normal distribution, related samples Wilcoxon signed rank test was used for the analysis. The significance level was set at 5%.

## Results

### Radiological assessment: pre-operatively

**Fig. 5:**
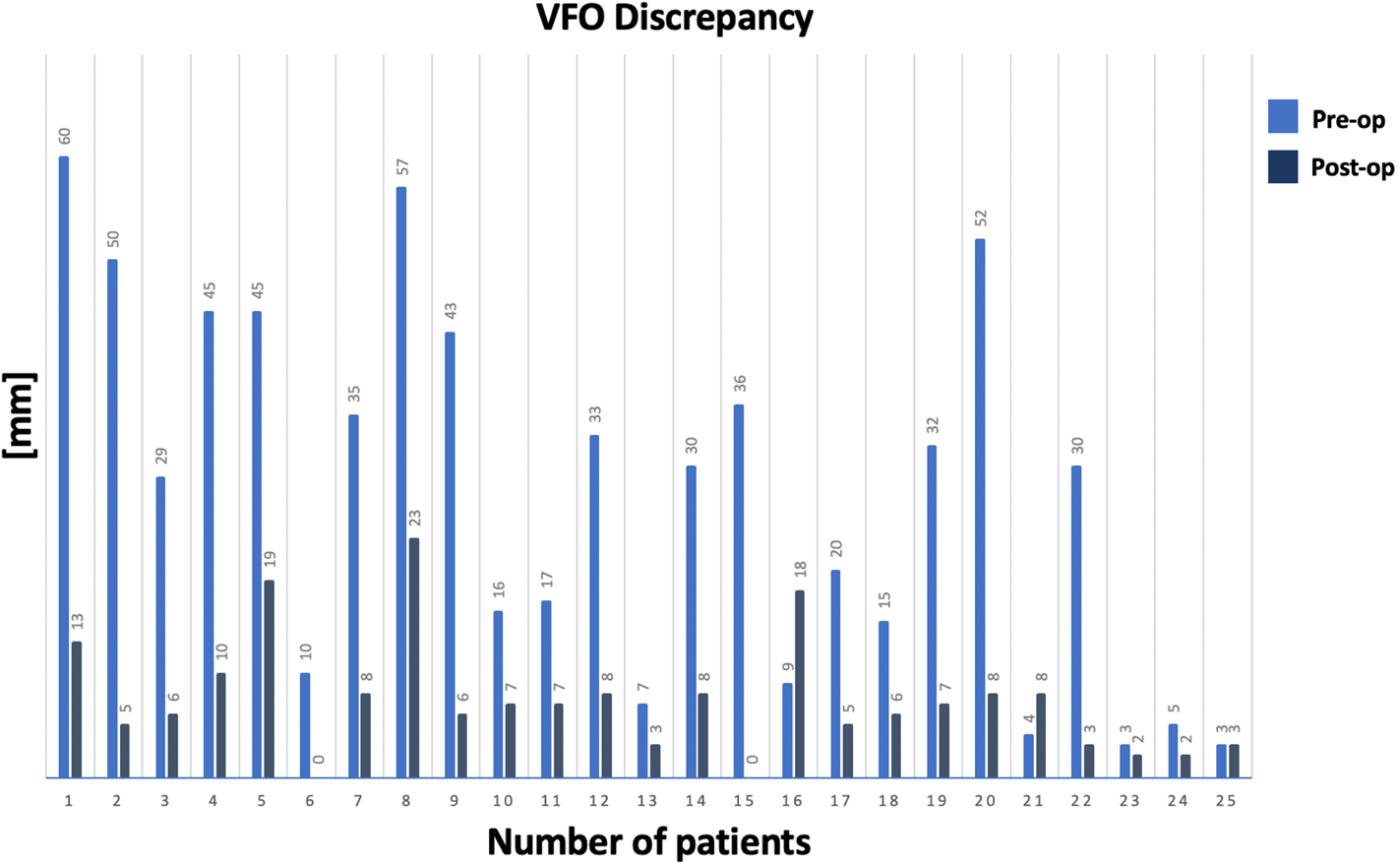
Column chart showing VOF discrepancy in all patients pre and post-operatively

The pre-operative VFO discrepancy between right and left leg was a mean of 28 mm (SD 17.99, min = 3, max = 60 mm). The surgical plan predicted a pelvic discrepancy between right and left leg (residual difference) of 3.08 mm on average (SD 6.9, min = 0, max = 27 mm), Fig. 5.

### Radiological assessment: post-operatively

**Fig. 6:**
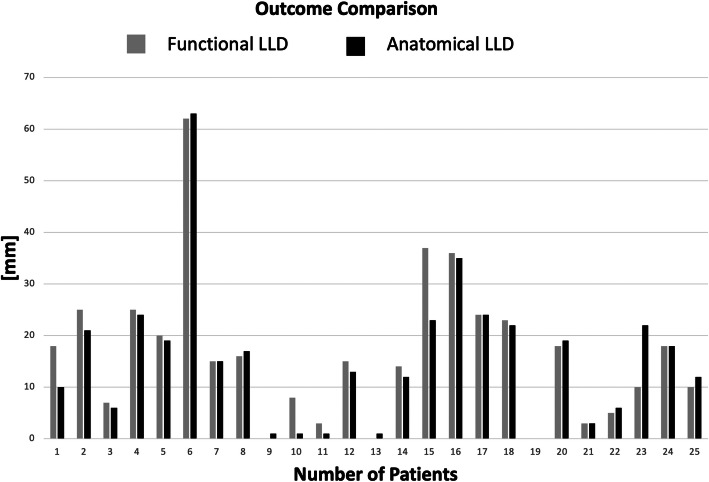
Column chart showing the functional LLD Vis-à-vis with the anatomical LLD for each case

Post-operatively, the VFO discrepancy between right and left leg was a mean of 7.4 mm (SD 5.7, min = 0, max = 23),

Functional LLD was a median 15 mm (IQR, 6 mm, 23.50 mm; min = 0, max = 62). Anatomical LLD was a median of 15 mm (IQR, 4.5 mm, 22 mm; min = 0, max = 63); Fig. 6. The difference was not statistically significant (*p* = 0.17).

**Fig. 7:**
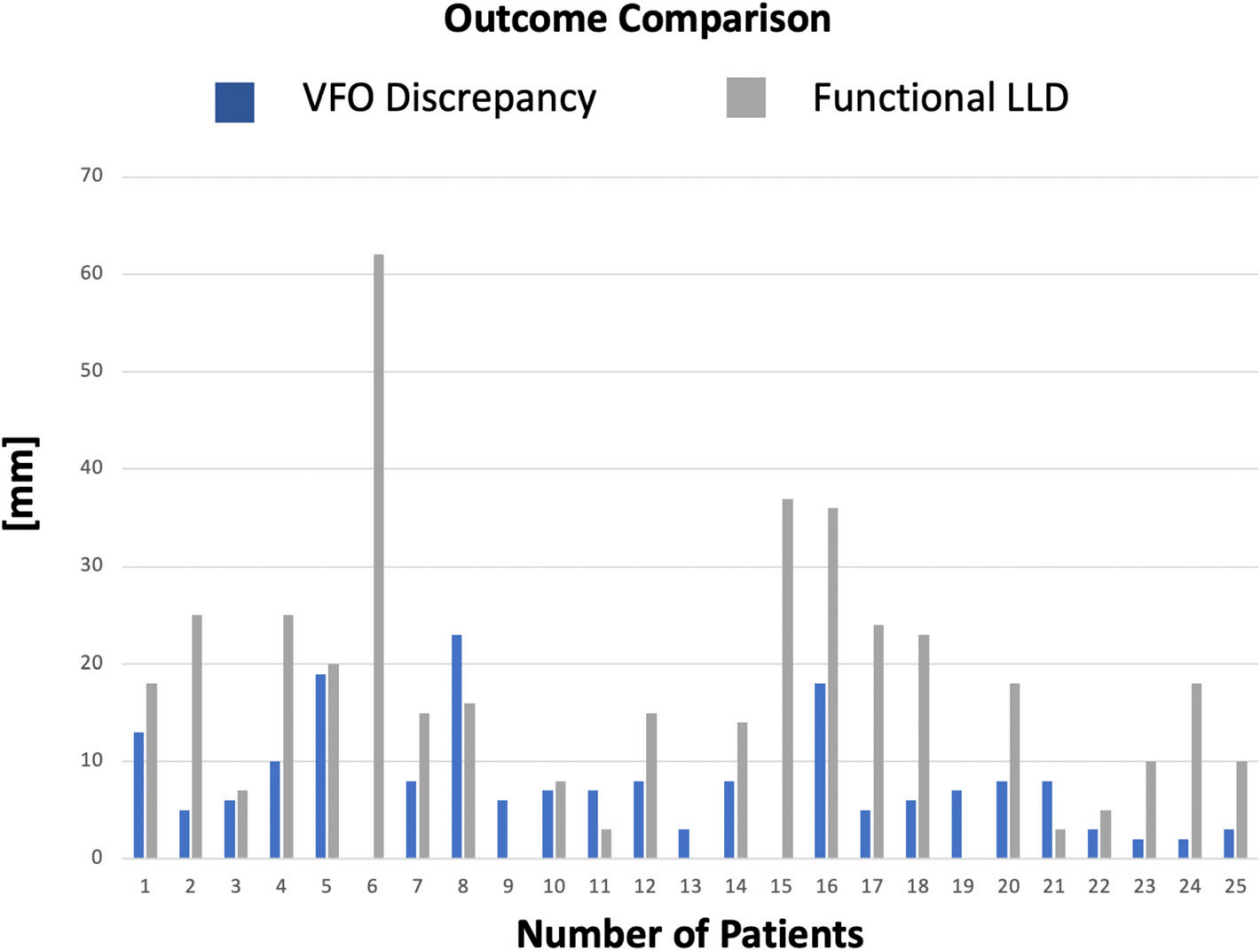
Column chart showing VFO discrepancy vis-à-vis with the functional LLD for each case

The difference between VFO discrepancy and functional LLD was found to be statistically significant, *p* = 0.0024 Fig. 7.

**Fig. 8:**
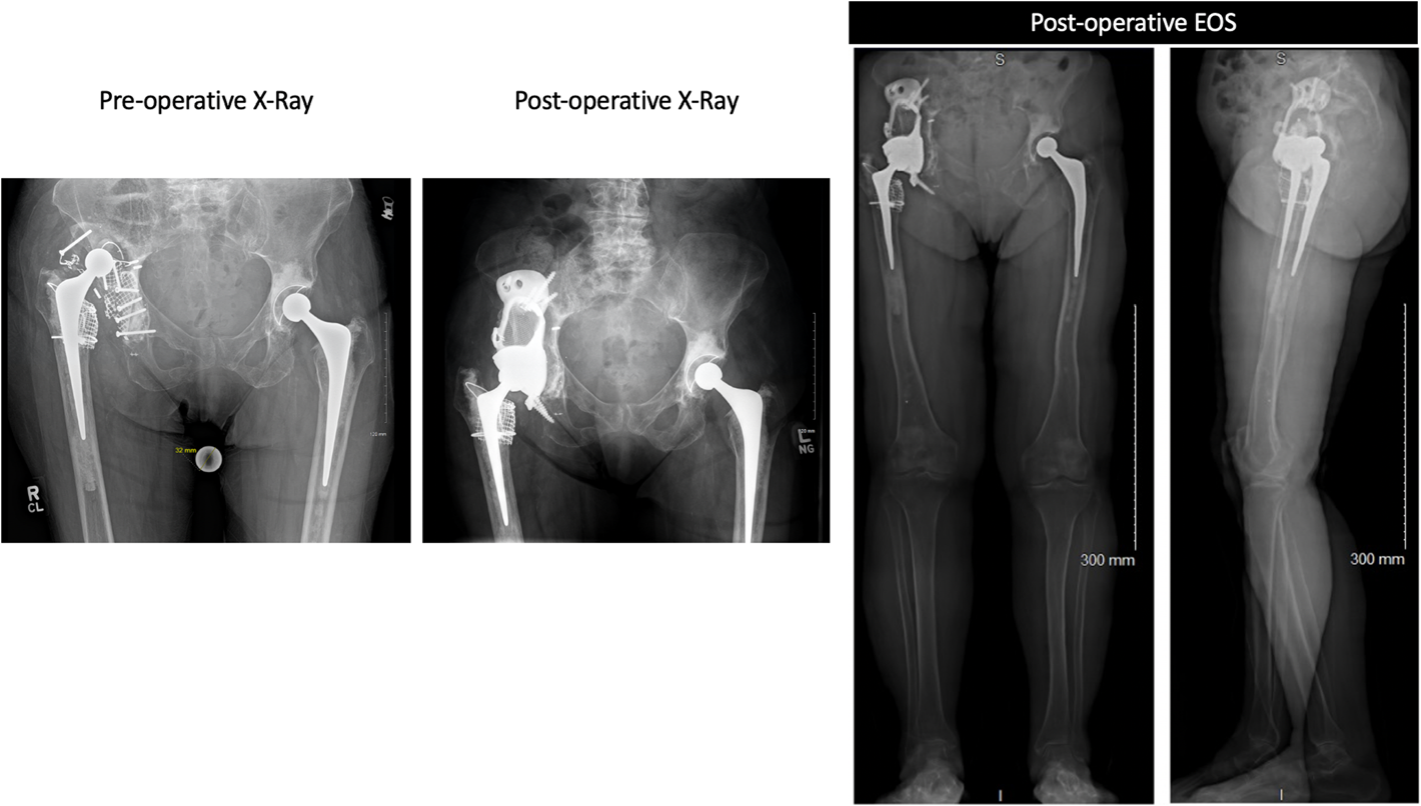
Case example. Pre-operative AP X-Ray (left image) showing major migration of the right hip, discrepancy between right and left VFO was 5 cm. Post-operative pelvic AP X-Ray (middle images) shows good restoration of centre of rotation, VFO discrepancy was 8 mm. Functional LLD measured on post-opeartive EOS imaging (right image) was 18 mm. Knee flexion can be observed on the lateral EOS image (left knee flexion =18°, right flexion =3°), difference in femur length = 2.5 cm

Nine cases (36%) showed a difference larger than 10 mm between VFO discrepancy and functional LLD. Length inequality, fixed flexion of the knee as well as and abduction deformity were present singularly of combined in these cases, Fig. 8.

#### Reproducibility

Intraclass correlation coefficient ICC showed excellent reproducibility between the two examiners for the measurement of post-operative CoR (ICC = 0.96, 95% CI, 0.90 to 0.98, *p* < 0.0001); for functional LL (ICC = 0.99, 95% CI, 0.997 to 0.994, *p* < 0.0001); and anatomical LL (ICC = 0.93, 95% CI, 0.83 to 0.97, *p* < 0.0001);

#### Clinical outcome

Duration of post-operative follow up and post-operative walking status are shown in Table 2. Mean oxford hip score at latest follow up was 32.1/48.

**Table 2:** Post-operative outcomes

Case	Follow-up time (months)	Post-operative walking aids	Complications
**1**	52	Unilateral walking stick	Nil
**2**	50	None	Transient foot drop
**3**	47	Unilateral walking stick	Nil
**4**	45	Unilateral crutch	Nil
**5**	48	None	Nil
**6**	45	Bilateral crutches, electric chair outside	Nil
**7**	45	Unilateral walking stick	Nil
**8**	35	Bilateral elbow crutches	Nil
**9**	37	None	Nil
**10**	38	None	Nil
**11**	34	Bilateral crutches	Nil
**12**	35	Bilateral crutches	Nil
**13**	29	Bilateral crutches	Nil
**14**	42	Unilateral walking stick	Nil
**15**	39	Walking crutches	Nil
**16**	22	Zimmer frame	Dislocation
**17**	29	Zimmer frame	Nil
**18**	25	Unilateral walking stick	Nil
**19**	24	None	Nil
**20**	26	Unilateral walking stick	Nil
**21**	24	Bilateral crutches	Nil
**22**	24	None	Nil
**23**	20	One crutch outdoors	Nil
**24**	26	Bilateral crutches	Nil
**25**	25	Bilateral crutches	Nil

## Discussion

In recent years, image analysis and 3D printing technology, in particular the use of patient-matched 3D printed cups, have allowed patients to walk again. LL equality is an important goal of any hip arthroplasty procedure as it affects functional outcome, and it is particularly challenging in revision surgery in presence of major complex lower limb deformity. Imaging of the pelvis and femurs is the gold standard used pre and post-operatively for planning and assessing limb length.

In planning revision surgery to design customised implants, the patients are CT scanned in supine position [16] and the true functional LL cannot be evaluated. Pelvic measurements of VFO constitute a surrogate for LL [23], but that can lead to significant limitations not taking into account any causes of LLD which do not involve the hip [24]; including abnormal pelvic orientation, bone length inequality, fixed flexion and asymmetrical femoral and tibial torsion [25]. LDBR, EOS, offers upright imaging and 3D reconstruction of the lower limbs to accurately measure LL with low radiation exposure and with negligible magnification errors [17].

We aimed to understand if 3D planning is effective at equalising the limbs when large acetabular defects are present, to measure the amount of CoR that can be brought down without compromising nerve functionality, in other words, if VFO is a good surrogate for LL in acetabular reconstruction.

We reported on the measurement of functional LL following reconstruction of significant acetabular defects. We found that post-operatively, the CoR discrepancy between right and left leg was of 7.4 mm on average (SD 5.7, min = 0, max = 23), therefore within the “safe” range of discrepancy [25–27]. There was a significant difference between the difference in right and left VFO and the measure of functional LLD. 36% of the cases (9/25) presented a difference larger than 10 mm between VFO discrepancy and functional LLD. Length inequality, fixed flexion of the knee as well as and abduction deformity were present singularly of combined in these cases.

No revision surgery has occurred to date, one patient dislocated the hip 6 months post-operatively. It was treated with one close reduction procedure with no further dislocation. One patient presented with a transient foot drop post-operatively which resolved over time. Mean oxford hip score at latest follow up was 32.1/48.

### Incidence, extent and impact of LLD after THA

LLD is defined as the inequality in the overall length of the right and left lower limb, it occurs in up to 70% of the entire population [28]. As many as one-third of healthy asymptomatic individuals have 5 mm to 2 cm of limb-length discrepancy and may not be aware of it. In fact, there are multiple potential compensatory mechanisms, such as toe-walking on the shorter side, circumduction and knee flexion of the longer side, or hyperpronation [25]. These compensatory mechanisms often act simultaneously to aid gait and may be present in patients with large acetabular defects before revision surgery.

LLD is classified as clinically relevant when it exceeds 20 mm [26]. Failure of hip implants, congenital conditions, tumour, trauma can lead to LL disparity and significant CoR displacement, often exceeding the level beyond which the risk of neurological damage is possible. LLD is associated with gait and posture abnormalities [6–8] and numerous clinically relevant issues, including arthritis of lower limbs and lumbar spine [7] as well as sciatic nerve damage [9].

It is unclear the extent of LL that can be safely restored after revision arthroplasty in patients with large acetabular defects. Complete equality between leg lengths is considered ambitious, a discrepancy of up to 10 mm has been generally set as an acceptable threshold in literature. However, this is not based on studies including severe acetabular bone loss.

For these cases, literature has reported LLD ranging from 3 to 70 mm, with an incidence of 1 to 17% after primary hip replacement [29] and 1 to 50% after total hip replacement in general [30]. The range of the average LLD reported after primary surgery, is between 3 and 17 mm; the range is 20 to 40 mm in case of large bony defects. Generally, a leg lengthening of more than 10 mm, is often observed after THA in half cases [29]. A clear distinction between acceptable and unacceptable LLD is yet to be defined.

LLD is negatively correlated with a high Oxford Hip Score (OHS), normal gait, body posture and patients perceiving LLD often complain for lower back pain, as well as, general dissatisfaction and they are commonly in need of shoe elevation [29]. These may lead to early dislocation or loosening, resulting in surgical failure and recurrence of surgery [30]. Clinically relevant issues, such as arthritis and sciatic nerve injury, have been also associated with leg discrepancies. Nerve palsy is correlated with the amount of lengthening performed during surgery, but there is no known safe threshold [25].

### Measuring LLD

Methods of correcting LLD include 2D or 3D pre-operative planning using X-Rays or CTs, intraoperative identification of anatomical landmarks and navigation techniques [24]. Computer-assisted navigation techniques rely on the usage of intraoperative landmarks. This method generally eliminates errors, but is considered an unwieldy and expensive tool, beside the steep learning curve. Pre-operative planning (2D) based on radiographs remains the gold standard, although of 2D nature and associated with magnification issues and therefore unreliable. CT-based 3D planning overcomes this at the expense of higher radiation dose. For this reason, CT acquisition has a limited field of view, it usually excludes images of full lower limbs.

### Comparison with previous studies

The range of pre-operative VFO discrepancy found in our study group aligns with what reported by Joshi et al. [31] documenting on the clinical outcome of using titanium cementless customised implants in patients with large acetabular deficiency. Mean pre-operative discrepancy was reported to be 35 mm (range 10-40 mm) and post-operative LLD 15 mm, with two patients having more than 20 mm discrepancy post-operatively. Garbuz et al. [5], who recorded the mid-term clinical outcome of allografts in patients with acetabular defects, reported a mean pre-operative LLD of 29 mm (range 10-60 mm) and a post-op LLD of 8 mm (range 0 to 30 mm), with 1 patient having more than 20 mm discrepancy. All measurements were performed on AP pelvic radiographs, thus with limited accuracy.

### Limitations

We acknowledge limitations. Firstly, pre-operative EOS imaging was not available for all the presented patients and therefore it was not possible to verify pre-existing conditions below the hip joint. We therefore excluded the measurement of pelvic orientation which affects LL but it is known to potentially vary after surgery. Secondly, we did not investigate the degree of any foot deformities radiographically as this would have been challenging to assess with the available data. Lastly, we acknowledge the small sample size of these extremely difficult cases, although our hospital constitutes the largest orthopaedic hospital in the UK, highlighting the rarity of their kind.

## Conclusions

This is the first study to investigate limb length discrepancy in functional position after reconstruction of large acetabular defects. VFO is not an optimal surrogate for LL when there is significant bone loss leading to length inequality, fixed flexion of the knee and abduction deformity.

Although challenging, LLD and gait abnormalities can be greatly improved with the aid of an accurate surgical planning. Surgeons and engineers should consider the integration of EOS imaging in surgical planning of reconstruction of large acetabular defects.

## Data Availability

The datasets used and/or analysed during the current study are available from the corresponding author on reasonable request.
